# Quiet ego is associated with positive attitudes toward Muslims

**DOI:** 10.3389/fpsyg.2022.893904

**Published:** 2022-08-02

**Authors:** Rosemary Lyn Al-Kire, Heidi A. Wayment, Brian A. Eiler, Kutter Callaway, Jo-Ann Tsang

**Affiliations:** ^1^Baylor University, Waco, TX, United States; ^2^Northern Arizona University, Flagstaff, AZ, United States; ^3^Davidson College, Davidson, NC, United States; ^4^School of Mission and Theology, Fuller Theological Seminary, Pasadena, CA, United States

**Keywords:** intergroup attitudes, quiet ego, intergroup relations, prejudice, authoritarianism, social dominance

## Abstract

Well-known predictors of prejudice toward Muslims include social dominance and authoritarianism. However, a gap exists for variables reflecting a rejection or mitigation of ideological motivations associated with prejudice toward Muslims. We examined if quiet ego was related to positive attitudes toward Muslims, and whether this could be explained by lower levels of authoritarianism, social dominance, and the motivation to express prejudice. We explored this possibility across two studies of adults in the United States (*N* = 376; *N* = 519). In Study 1, regression results showed quiet ego was directly associated with positive attitudes toward Muslims. Study 2 utilized path analyses and found that the direct relationship between quiet ego and positive attitudes toward Muslims was explained by associations between quiet ego and lower endorsement of authoritarianism, social dominance, and the internal motivation to express prejudice toward Muslims. Moreover, these associations held when accounting for several correlates of intergroup attitudes.

## Introduction

With the rise of globalization, interactions with dissimilar others have become a part of daily life, particularly in increasingly diverse countries such as the United States. For example, although the United States is comprised of predominantly Christians, the Muslim population has grown from 2.35 million in 2007 to 3.45 million in 2017. This growth is expected to continue. For example, by 2040, Muslims are projected to outnumber the percent of American Jews (Pew Research Center, 2018). Beyond demographic changes, the United States is experiencing a time of increased sociopolitical division, including between religious groups (Haidt, 2013). With this rise, there has also been a subsequent increase in ethnocentrism and hostility amongst majority groups toward marginalized minority groups, such as immigrants, refugees, and Muslims. For example, in 2017, hate crimes and assaults against Muslims in the United States rose to the highest level seen since the September 11, 2001 attacks in the United States (Pew Research Center, 2017). This bias is also mirrored in U.S. political policies. For example, in early 2017, President Trump issued an executive order which intentionally banned travelers from seven Muslim-majority countries, claiming they posed a heightened terrorist threat, thus reinforcing Islamophobic beliefs. Justifications for this policy included narratives of intergroup threat and competition, which underlie two major ideologies commonly associated with prejudiced attitudes (Duckitt, 2006).

Given the prevalence and impact of prejudice toward Muslims, identification of modifiable factors which may be associated with positive attitudes toward Muslims and other marginalized groups is needed. Recent shifts in psychology and research on human flourishing call for researchers to identify ways to nurture positive characteristics as well as prevent or combat negative phenomena such as prejudice. A promising construct that may be related to positive intergroup attitudes is quiet ego (Wayment and Bauer, 2017a,b). Specifically, we argue that quiet ego should be negatively associated with motivational and ideological bases of prejudice toward Muslims. The goal of the current research was to examine whether quiet ego would be directly associated with positive attitudes toward Muslims, as well as indirectly *via* attenuated psychological indicators of prejudice.

### Quiet ego as a predictor of positive intergroup attitudes

Quiet ego refers to a way of construing a self-identity that reflects eudaimonic principles characterized by a motivation to balance self-and other-concerns (Wayment and Bauer, 2017a). Individuals who score higher in quiet ego report greater well-being outcomes even while accounting for mindfulness, self-compassion, and authenticity (Wayment et al., 2015). Previous work shows that quiet ego is positively associated with universalism, benevolence, and self-direction values (Wayment and Bauer, 2017a). Further, quiet ego was recently identified as a strong correlate of the light triad (i.e., Kantianism, humanism, and faith in humanity), described as a loving and beneficent orientation towards others (Kaufman et al., 2019). Reflecting an orientation that is largely non-judgmental and, thus, tolerance-prone, quiet ego is related to traits, such as honesty-humility, conscientiousness, agreeableness, and openness to experience (Wayment et al., 2015), which have been identified as relevant traits in the link between personality and intergroup attitudes (Sibley et al., 2010).

We suggest that quiet ego may be a particularly strong predictor of positive intergroup attitudes. As a higher-order construct, quiet ego reflects the intersection of four psychological characteristics: perspective taking, inclusive identity, detached awareness, and growth-mindedness (Wayment et al., 2015). Each of these dimensions can be linked to positive intergroup attitudes. For example, perspective-taking is associated with an increase in empathy for others and is a key predictor of intergroup attitudes (see Aronson et al., 1978; Galinsky and Moskowitz, 2000; Galinsky and Ku, 2004; Pettigrew and Tropp, 2006; Stewart et al., 2010). Additionally, inclusive identity calls us to focus on similarities with others, an essential process of empathy (Brewer and Pierce, 2005; Kaufman et al., 2019). This relates to work which finds that fostering a superordinate or common identity is also associated with decreases in intergroup biases (see Dovidio et al., 2003 for a review). Finally, detached awareness, or mindfulness, along with growth mindedness, may relate to greater internal motivations to respond without prejudice (Gervais and Hoffman, 2013). A recent meta-analysis identified a small but robust effect of mindfulness on positive intergroup attitudes (Oyler et al., 2021). Importantly, mindfulness is characterized by self-focus, whereas quiet ego is reflected in a balanced focus on the self and others, suggesting the effect of quiet ego on positive intergroup attitudes may be greater than mindfulness itself. Consistent with this idea, a recent study found experimentally induced state mindfulness reduced guilt which thereby reduced prosocial reparation (Hafenbrack et al., 2022). Altogether, this evidence suggests quiet ego as the intersection of these four psychological characteristics may be a strong source of positive intergroup attitudes. Given these demonstrated connections, we also theorize that the relation between quiet ego and attitudes toward Muslims is indirect, such that quiet ego is associated with ideological motivations which underlie prejudice. To unpack this idea further, we turn to previous work on ideology and prejudice.

### Ideological and motivational dimensions of prejudice toward Muslims

The dual process model of ideology and prejudice (Duckitt, 2001) is a useful framework for understanding antecedents of prejudiced attitudes. In this model, right wing authoritarianism (RWA) and social dominance orientation (SDO) are used to explain how prejudiced attitudes emerge through personality, values, and worldviews (Sibley and Duckitt, 2008; Duckitt and Sibley, 2010). RWA and SDO are both influenced by personality characteristics, context, and worldview beliefs (Duckitt and Sibley, 2017), but reflect ideological dimensions rather than personality itself. For example, SDO is characterized by the endorsement of hierarchical structures that shape and uphold group-based prejudice (Pratto et al., 2006), and is rooted in values of power, achievement, and a competitive worldview (Duckitt and Sibley, 2017). Additionally, RWA is characterized by adherence to social authorities, traditions, and norms, and is rooted in perceptions of threat (Lee et al., 2010). Although these variables remain relatively stable over time, and are partially heritable (Kleppestø et al., 2019), they are influenced by contextual factors, and thus amenable to change. For example, Dhont and colleagues found reduced levels of SDO after positive intergroup contact, both in an intervention and longitudinal sample of U.S. adults (Dhont et al., 2014). Importantly, researchers find support for a bidirectional relationship between SDO and empathy (a key aspect of quiet ego) over time (Sidanius et al., 2013), suggesting that quiet ego characteristics may also be related to SDO. Thus, a way in which quiet ego may be associated with positive attitudes toward Muslims is through a negative association with key motivational and ideological variables, each of which is associated with negative intergroup attitudes, namely RWA, SDO, and the internal and external motivations to express prejudice (MTEP).

Much of the variance in negative attitudes toward Muslims and other minoritized and marginalized groups can be explained by SDO and RWA (Rowatt et al., 2005). RWA and SDO are often analyzed in conjunction, given their robust but unique associations with prejudiced attitudes toward different outgroups (Duckitt, 2001). For example, SDO is more commonly associated with prejudice toward lower-status groups such as immigrants, whereas RWA is associated with prejudice toward groups perceived as threatening or norm-violating such as feminists (see Duckitt and Sibley, 2017 for a review). Together, these variables have been established as important for predicting a variety of prejudiced attitudes (Rowatt et al., 2005; Sibley and Duckitt, 2008; Hall et al., 2010; Brandt and Van Tongeren, 2017; Matić et al., 2019), and both are implicated in prejudice toward Muslims (Rowatt et al., 2005; Beck and Plant, 2018; Dunwoody and McFarland, 2018).

Furthermore, evidence from longitudinal studies supports a causal relationship between RWA, SDO, and prejudice (Sibley and Duckitt, 2013); suggesting these constructs may be a promising route to promote more positive intergroup attitudes. Together, RWA and SDO capture a substantial proportion (often more than half) of the variance in prejudice (Ekehammar et al., 2004; Sibley et al., 2006; Sibley and Duckitt, 2008) and to date, no other psychological factors have emerged which substantially contribute to the prediction of prejudice (Duckitt and Sibley, 2017). Furthermore, although it is uncommon for individuals to score highly on both SDO and RWA, those who do demonstrate the largest capacity for prejudice (Altemeyer, 2004).

Importantly, prejudiced attitudes captured by RWA and SDO do not capture motivation or intentionality to express prejudice; rather, RWA and SDO reflect more general ideological attitudes. To understand more deliberate forms of prejudice (i.e., hate crimes or speech) recent research has turned to a related construct—the motivation to express prejudice (Forscher et al., 2015), which captures both internal (e.g., personal values of treating people equally) and external (e.g., desire to avoid social backlash for expressing prejudice) motivations. More specifically, MTEP reflects both motivational deficiencies in responding without prejudice, coupled with the desire to express prejudicial attitudes. For example, someone with a high internal motivation to express prejudice toward Muslims might hold a personal belief that Muslims are inherently violent. Conversely, one with a high external motivation to express prejudice toward Muslims may show prejudice due to fear of backlash from their community if they were not to do so. Previous research shows that those high in both internal and external motivation show an increased likelihood of expressing prejudice (Plant and Devine, 1998). Given that the expression of prejudice influenced by RWA and SDO does not reflect prejudice motivations themselves, MTEP may shed light on the most aggressively prejudiced individuals, particularly toward groups that are both low in social status and are perceived as threatening. Furthermore, quiet ego’s balanced focus on the self and others may help mitigate these internal and external motivations to express prejudice.

## The current research

**Figure 1:**
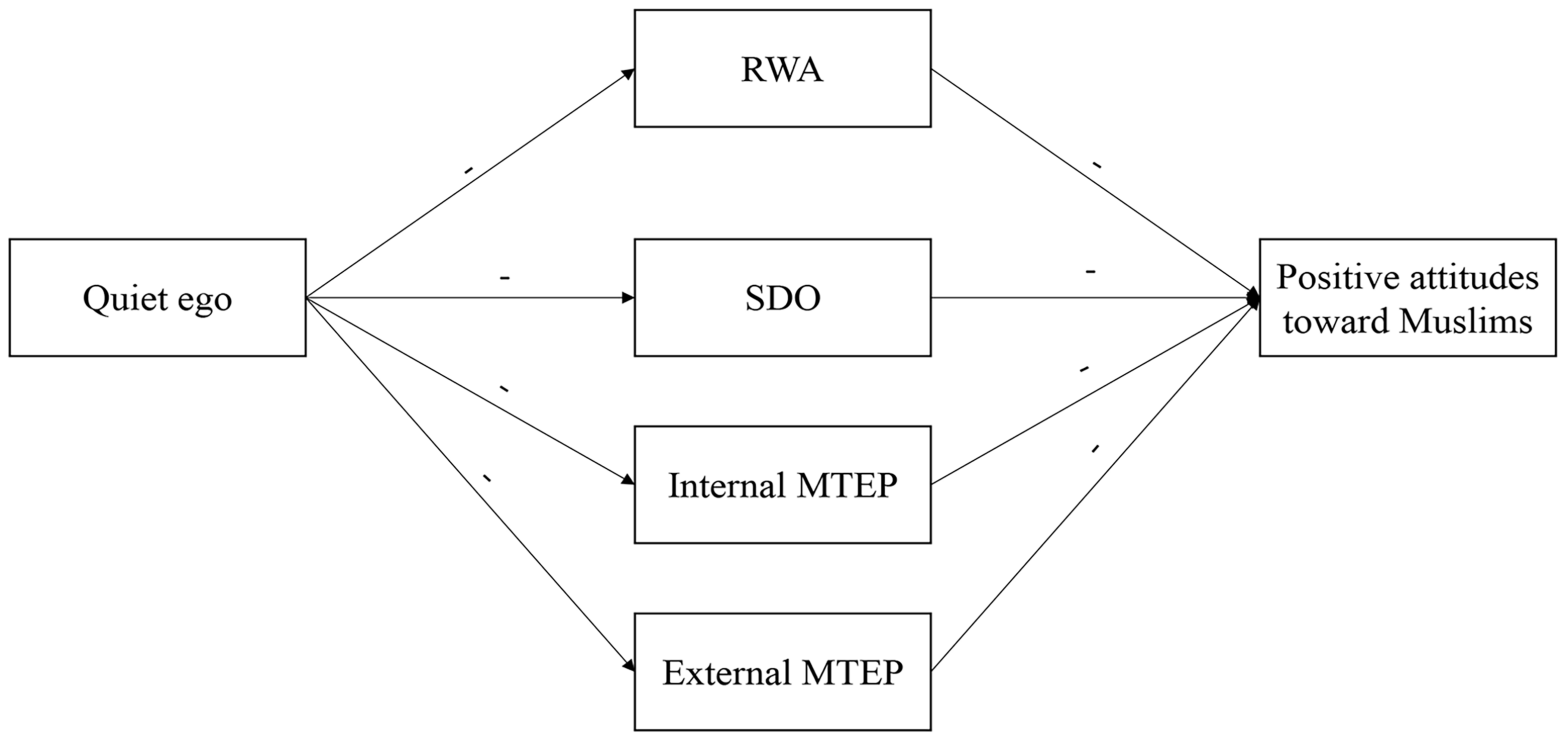
Hypothesized mediation model depicting expected paths between variables. Paths denoted by a “-” denotes an expected negative association.

In this work, we had two overarching research aims. The first was to test whether quiet ego was associated with positive attitudes toward Muslims in the United States. The second aim was to test whether this relationship could be explained by a lower endorsement of four well-known orientations and motivations toward prejudice: RWA, SDO, and internal and external MTEP. Figure 1 depicts the hypothesized model. In this model, we hypothesized that quiet ego would be negatively associated with RWA, SDO, and internal and external MTEP. In turn, RWA, SDO, and internal and external MTEP would be negatively associated with positive attitudes toward Muslims, indirectly accounting for the relationship between quiet ego and positive attitudes toward Muslims. We approached these questions through two studies: first, testing the direct effect of quiet ego on positive attitudes toward Muslims in Study 1, then by testing the indirect effect in Study 2.

## Study 1

### Method

#### Participants and design

Four hundred US-based participants were recruited from Cloud Research. Prior to data analysis, data were cleaned using predetermined data exclusions. Participants who completed less than half of the survey were removed from the dataset (*n* = 24). All participant IP addresses were then run through^1^ to ensure participants were from the United States (*n* = 21 excluded). Additionally, participants who failed the attention check items were excluded from analyses (*n* = 16). The final sample was comprised of 339 individuals (*M_age_* = 43.81, *SD_age_* = 15.19; 54.0% female). Of these, 71.3% were White, 11.7% Black or African American, 6.5% Asian or Pacific Islander, 5.6% Hispanic, 3.2% Native American, and 1.2% multi-racial. Additionally, participants were 47.5% Catholic, 34.9% Protestant, 10.6% other religions, 3.2% no religion, 1.8% Agnostic, and 1.5% Jewish. No participants in the final sample identified as Muslim. All study procedures were approved by the university’s institutional review board prior to data collection, and APA ethical standards were followed for all study-related activities. For both studies, all data exclusions and manipulations are reported, and analyses were conducted using all available data for each participant.

### Measures

#### Quiet ego

To measure quiet ego characteristics, participants completed the 14-item Quiet Ego Scale (Wayment et al., 2015). Participants rated items such as “For me, life has been a continuous process of learning, changing, and growth” and “I do jobs or tasks automatically, without being aware of what I am doing” on a five-point Likert-type scale ranging from 1 (*strongly disagree*) to 5 (*strongly agree*). Items were averaged to create a mean score, and the scale demonstrated good reliability in this sample (*α* = 0.79).

#### Attitudes toward Muslims

Attitudes toward Muslims were assessed with a single thermometer-style item. Participants were asked to rate a series of religious (e.g., Hindus, Muslims, and Christians), social (e.g., immigrants and heterosexuals), and ethnic-racial (e.g., Whites, Blacks, and Hispanics) groups on a slider scale ranging from 0 (*cold*) to 100 (*warm*). Only the thermometer for Muslims is reported for the purposes of this study.

#### Control variables

To understand the unique variance quiet ego captures in attitudes toward Muslims, we controlled for several known predictors of prejudice toward Muslims and other minoritized groups: political conservatism, religious interest, religious affiliation, and race (Strabac and Listhaug, 2008; Rowatt et al., 2009; Johnson et al., 2012).

##### Conservatism

A single item was used to assess political conservatism: “How would you describe yourself politically?” Participants responded on a scale of 1 (*very liberal*) to 7 (*very conservative*).

##### Religious interest

Religious interest has been used as a proxy of intrinsic religiousness, which is typically associated with more positive intergroup attitudes (Hunsberger and Jackson, 2005). A single item was used to assess religious interest: “How interested are you in religion?” Participants responded on a scale of 1 (*not at all interested*) to 9 (*extremely interested*).

##### Religious affiliation

A single item was used to assess religious attendance: “With which religion do you identify?” For the purposes of the analyses in this study, we dichotomized this into a single index (−0.5 = non-religious, 0.5 = religious).

##### Race

For the purposes of the analyses below, self-reported racial demographics were dichotomized into a single index (−0.5 = non-White, 0.5 = White).

### Results and discussion

To estimate the relation between quiet ego and positive attitudes toward Muslims, we regressed perceived warmth toward Muslims on quiet ego. Correlations and descriptive statistics are reported in Table 1. Additionally, to test the robustness of this effect, we tested a secondary model controlling several demographic correlates of prejudice: race, political conservatism, religious interest, and religious affiliation. Results from a *post-hoc* power analysis conducted in the pwr2ppl package in R (Aberson, 2019) revealed this sample provided greater than 99% power to detect an effect using correlations obtained from our data (see Table 1).

**Table 1:** Means, SDs, and correlations with CIs.

S.no	Variable	*M*	*SD*	1	2	3	4	5
1.	Quiet ego	3.63	0.55					
2.	Attitudes toward Muslims	56.00	31.24	0.39^**^ [0.30, 0.47]				
3.	Religious interest	6.67	2.10	0.13^**^ [0.03, 0.23]	0.12^*^ [0.02, 0.22]			
4.	Religious affiliation	0.46	0.20	0.05[−0.05, 0.15]	0.07 [−0.03, 0.17]	0.28^**^ [0.18, 0.37]		
5.	Conservatism	4.48	1.88	−0.13^*^ [−0.23, −0.03]	−0.25^**^ [−0.34, −0.15]	0.30^**^ [0.20, 0.39]	0.11^*^ [0.01, 0.21]	
6.	Race	0.22	0.45	0.01 [−0.09, 0.11]	−0.06 [−0.16, 0.04]	−0.10 [−0.20, 0.00]	0.00 [−0.10, 0.11]	0.04 [−0.06, 0.15]

*M* and *SD* are used to represent mean and standard deviation, respectively. Values in square brackets indicate the 95% CI for each correlation. The CI is a plausible range of population correlations that could have caused the sample correlation (Cumming, 2014).

* indicates *p* < 0.05;

** indicates *p* < 0.01.

Consistent with our hypothesis, quiet ego was associated with greater warmth toward Muslims, *b* = 23.35, *t*(338) = 8.24, *p* < 0.001. In Model 2, which tested the robustness of this effect, this association remained significant with race, conservatism, and religiosity included as covariates (see Table 2).^2^

**Table 2:** Regression results for Study 1.

Variable	*B* (95% CI)	*β*	*t*	*sr^2^*	*R^2^*
**Step 1**					0.15
Quiet ego	21.93 (16.66, 27.20)	0.39	8.19^***^	0.15	
**Step 2**					0.22
Quiet ego	18.99 (13.75, 24.22)	0.34	7.14^***^	0.11	
Race	−2.80 (−9.10, 3.50)	−0.04	−0.87	0.00	
Conservatism	−4.13 (−5.73, −2.52)	−0.25	−5.06^***^	0.05	
Religious affiliation	6.07 (−8.06, 20.19)	0.04	0.84	0.00	
Religious interest	2.04 (0.54, 3.53)	0.14	2.68^**^	0.02	

** *p* < 0.01;

*** *p* < 0.001.

Results from Study 1 indicate that quiet ego is positively associated with perceptions of warmth, or positive attitudes, toward Muslims, and this effect was robust to the inclusion of several demographic covariates associated with prejudice. What remains unclear are the mechanisms by which quiet ego is associated with attitudes toward Muslims. To test this in Study 2, we examined the indirect relation between quiet ego and attitudes toward Muslims through several well-documented predictors of outgroup attitudes: RWA, SDO, and internal and external MTEP. Additionally, in Study 1, we measured attitudes toward Muslims using a single item index which might not have captured the nuance in attitudes toward Muslims. In Study 2, we sought to test the replicability of this effect using a more nuanced multi-item measure of attitudes toward Muslims.

## Study 2

In Study 2, we sought to replicate and extend Study 1 findings and tested four potential mechanisms by which quiet ego may be associated with attitudes toward Muslims: RWA, SDO, and internal and external MTEP. We hypothesized that quiet ego would be positively associated with positive attitudes toward Muslims, conceptually replicating results from Study 1 using a more nuanced multi-item measure of attitudes toward Muslims and controlling for socially desirable responding. Additionally, we hypothesized quiet ego would be negatively associated with RWA, SDO, and internal and external MTEP. Finally, we predicted that the association between quiet ego and positive attitudes toward Muslims would be explained by four ideological and motivational variables associated with outgroup attitudes, namely RWA, SDO, and internal and external MTEP.

### Method

#### Participants

Participants were recruited from introductory psychology courses from a southwestern university over the course of two semesters and earned partial course credit for their participation. Participants (*N* = 519; 76.9% female, 21.0% male, 2.2% missing, or other) reported their religious affiliation as 56.5% Christian, 13.3% Agnostic, 12.9% Atheist, 6.9% missing or other, 6.7% Spiritual, 1.3% Native American Church, 0.8% Buddhist, and 0.4% Muslim^3^; ages ranged from 18 to 32 years, with a mean age of 19.16 (*SD* = 1.54). Racial and ethnic demographic information for these samples were not collected; however, previous samples from this population have reported as follows: 75% White/Caucasian, 21% Hispanic, 8% Black/African American, 4% Native American, 2–3% Pacific Islander, and 1–2% Other. All study procedures were approved by the university’s institutional review board prior to data collection, and APA ethical standards were followed for all study-related activities. Results from a *post-hoc* power analysis conducted in the pwr2ppl package in R (Aberson, 2019) revealed this sample provided greater than 99% power to detect an indirect effect for all three mediators using correlations obtained from our data (see Table 3).

**Table 3:** Means, SDs, and correlations with CIs for Study 2.

S.no	Variable	*M*	*SD*	1	2	3	4	5	6	7
1.	Quiet ego	3.61	0.45							
2.	RWA	3.37	0.81	−0.30^**^ [−0.38, −0.22]						
3.	SDO	2.33	1.15	−0.52^**^ [−0.58, −0.45]	0.53^**^ [0.47, 0.59]					
4.	External MTEP	2.07	1.56	−0.38^**^ [−0.45, −0.31]	0.42^**^ [0.35, 0.49]	0.56^**^ [0.50, 0.62]				
5.	Internal MTEP	1.98	1.65	−0.39^**^ [−0.46, −0.31]	0.39^**^ [0.31, 0.46]	0.56^**^ [0.49, 0.61]	0.93^**^ [0.92, 0.94]			
6.	Ideology	35.19	25.46	−0.07 [−0.16, 0.02]	0.28^**^ [0.20, 0.36]	0.22^**^ [0.13, 0.30]	0.12^*^ [0.03, 0.20]	0.10^*^ [0.02, 0.19]		
7.	SDR	3.27	0.67	0.12^**^ [0.03, 0.20]	0.13^**^ [0.04, 0.21]	−0.03 [−0.11, 0.06]	−0.01 [−0.10, 0.08]	0.01 [−0.07, 0.10]	0.04 [−0.04, 0.13]	
8.	ATM	4.51	0.81	0.37^**^ [0.29, 0.44]	−0.57^**^ [−0.62, −0.51]	−0.66^**^ [−0.71, −0.61]	−0.60^**^ [−0.65, −0.54]	−0.60^**^ [−0.65, −0.54]	−0.28^**^ [−0.36, −0.20]	−0.01 [−0.10, 0.07]

RWA, right wing authoritarianism; SDO, social dominance orientation; MTEP, motivation to express prejudice; ATM, positive attitudes toward Muslims; and SDR, socially desirable responding. Values in square brackets indicate the 95% CI for each correlation. The CI is a plausible range of population correlations that could have caused the sample correlation (Cumming, 2014).

* indicates *p* < 0.05;

** indicates *p* < 0.01.

#### Materials and measures

##### Political orientation

Political orientation was assessed by asking participants to rate their political standing on a sliding scale from 1 (*very left wing*) to 100 (*very right wing*).

##### Right wing authoritarianism

The Right-Wing Authoritarianism Revised Scale (Rattazzi et al., 2007) was used to measure RWA. The scale is comprised of 15 items and included statements such as, “The majority of those who criticize proper authorities in government and religion only create useless doubt in people’s mind” and “What our country needs most is disciplined citizens, following national leaders in unity.” Participants rated their agreement with each statement on a scale of 1 (*totally disagree*) to 7 (*totally agree*). A composite score was calculated, with a higher score reflecting a higher degree of RWA. Scale reliabilities were good (*α* = 0.80).

##### Social dominance orientation

The 16-item Social Dominance Orientation Scale (Pratto et al., 1994) was used to assess SDO. Sample items included “Some groups are simply inferior to other groups” and “It’s OK if some groups have more of a chance in life than others.” Participants were instructed to rate each item on a scale of 1 (*very negative*) to 7 (*very positive*). A mean score was calculated, with higher scores reflecting a higher level of social dominance orientation. Reliability was excellent in this sample (*α* = 0.94).

##### Quiet ego

The same 14-item Quiet Ego Scale was used as in Study 1. Scale reliability was good in this sample (*α* = 0.76).

##### Motivation to express prejudice

The Motivation to Express Prejudice Scale (Forscher et al., 2015) was adapted from its original form (which assessed prejudice toward African Americans) to assess motivation to express prejudice towards Muslims. The scale is composed of 12 items assessing both internal-personal motivations as well as external-social motivations. Participants were asked to rate their agreement with each statement using a Likert-type scale ranging from 1 (*strongly disagree*) to 9 (*strongly agree*). Example items include “According to my personal values, expressing positive feelings about Muslims is wrong” (internal), and “I express negative thoughts about Muslims to avoid negative reactions from others” (external). A composite score was calculated, with a higher score reflecting a higher motivation to express prejudice toward Muslims. Reliability was excellent in this sample for both internal (*α* = 0.97) and external (*α* = 0.94) motivation to express prejudice.

##### Attitudes toward Muslims

The Attitudes toward Muslims Scale (Altareb, 1997) was used to analyze explicit prejudice toward Muslims. The 30-item inventory assessed five dimensions of positive attitudes toward Muslims. These factors were defined as: Positive Feelings about Muslims (e.g., “Muslims are friendly people”; α = 0.94), Muslims as Separate or Other [e.g., “I would support a measure deporting Muslims from America” (reverse-scored); α = 0.83], Restriction of Personal Choice/Freedom [e.g., “Muslims are strict” (reverse scored); α = 0.75], Fear of Muslims [e.g., “Muslims should be feared” (reverse scored); α = 0.68], and Dissimilarity of Muslims [e.g., “The Muslim religion is too strange for me to understand” (reverse scored); α = 0.54]. Participants were instructed to respond to items on a scale of 1 (*strongly disagree*) to 6 (*strongly agree*). Overall scores were calculated such that a higher composite score on this measure was interpreted as more positive attitudes toward Muslims. Reliability for the full scale was excellent (α = 0.92). In support of the validity of this measure, this measure was positively correlated with general warmth toward Muslims [measured by a single thermometer item from 0 (*cold*) to 100 (*warm*)], *r* = 0.50, *p* < 0.001.

##### Socially desirable responding

Explicit prejudice may be susceptible to socially desirable response bias, and is often controlled for in studies of prejudice. To control for this in our model, we included the Balanced Inventory of Desirable Responding Short Form (BIDR-16; Hart et al., 2015). Participants responding to items such as “I never regret decisions” and “I never cover up mistakes” on a Likert-type scale ranging from 1 (*strongly disagree*) to 7 (*strongly agree*). This measure demonstrated good reliability (α = 0.82).

### Results

#### Mediation analyses

A parallel multiple mediation model was conducted using the PROCESS add-on for SPSS (Hayes, 2017) to test the hypothesized model (see Figure 1). Completely standardized indirect effects were estimated using bootstrapped estimates with 10,000 resamples and 95% bias-corrected (BC) CIs. Statistical significance of the indirect effect was inferred if the CI did not contain zero. Completely standardized regression coefficients are reported for each path in the models reported below. Descriptive statistics are reported in Table 3.

**Figure 2:**
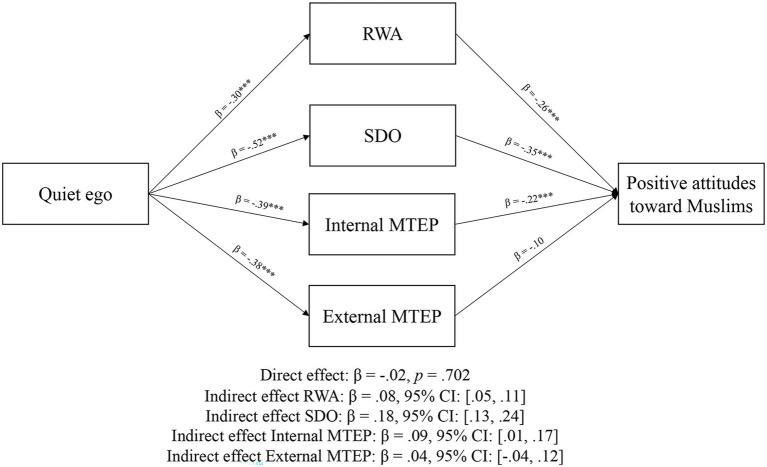
Mediation results from Study 2. ***p < 0.001.

**Figure 3:**
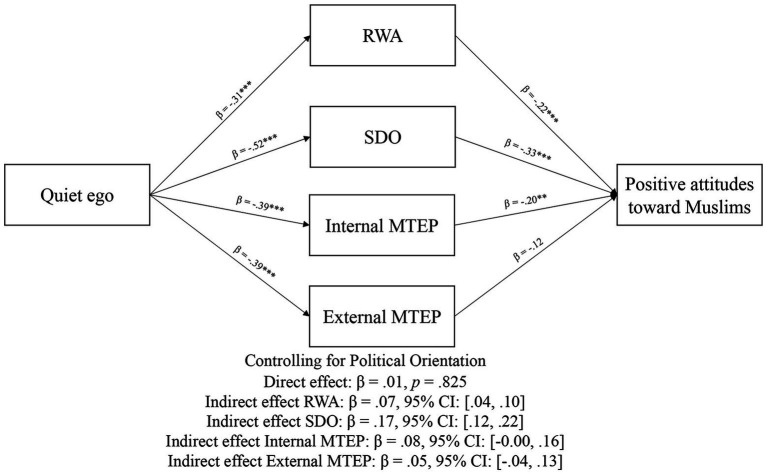
Mediation results from Study 2, controlling for political orientation. **p < 0.01; and ***p < 0.001.

**Figure 4:**
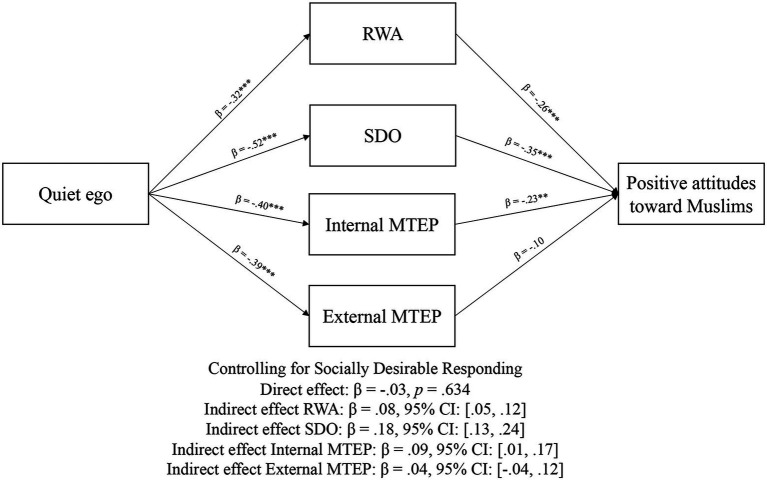
Mediation results from Study 2, controlling for socially desirable responding. **p < 0.01; and ***p < 0.001.

Figure 2 shows the results of the parallel multiple mediation models. All a and b paths were significant (*p*s < 0.001). As indicated by the nonsignificant direct effect, and significant indirect effect, the relationship between quiet ego characteristics and positive attitudes toward Muslims was mediated by RWA, SDO, and internal MTEP, but not external MTEP. We ran a second model with political orientation as a covariate, and results were consistent with model 1 (see Figure 3). Finally, we ran a third model with socially desirable responding as a covariate (see Figure 4). As hypothesized, the association between quiet ego and positive attitudes toward Muslims was mediated by RWA, SDO, and internal MTEP across all three models; however, the hypothesized indirect path for external MTEP was non-signfiicant.

## General discussion

Across two studies, we examined the relationship between quiet ego and attitudes toward Muslims. In Study 1, we tested the direct effect of quiet ego on positive attitudes toward Muslims and tested potential mechanisms of this relationship in Study 2. We hypothesized that quiet ego would be associated with more positive attitudes toward Muslims, and this relationship would be explained by lower levels of motivational orientations toward prejudiced attitudes (i.e., RWA, SDO, and internal and external MTEP). Results from these studies support the hypotheses that quiet ego is both directly associated with more positive attitudes toward Muslims (Study 1), and indirectly through RWA, SDO, and internal MTEP (Study 2). Further, we found that these relations held when controlling for demographic covariates (Study 1) as well as political orientation and socially desirable responding (Study 2), suggesting our predicted associations are robust to demographic and psychological correlates of intergroup attitudes. These results are consistent with the dual process model of prejudice, which posits that RWA and SDO are influenced by personality and situational characteristics, such as quiet ego. Below, we discuss possible explanations for these results as well as implications, strengths, limitations, and recommendations for future research.

The identified associations between SDO, RWA, and quiet ego contribute to the broader understanding of quiet ego and related processes. Given that SDO is characterized by a preference for group-based inequality and RWA is characterized by adherence to traditionalism and group norms, quiet ego characteristics may reflect a general ability to think less hierarchically about individuals and groups and demonstrate higher socio-cognitive flexibility. Supporting this idea, previous studies found quiet ego was consistently associated with less rigid and more pluralistic thinking (Wayment et al., 2015).

Aside from an association with a lower cognitive inclination toward prejudiced attitudes (i.e., RWA and SDO), quiet ego characteristics were also associated with decreased internal and external motivations to express prejudice (MTEP). Given the importance of universalism and benevolence values in the quiet ego construct (Wayment and Bauer, 2017a), quiet ego characteristics could aid in facilitating motivations to promote positive rather than negative intergroup attitudes. There is some existing precedence for this process for prejudice reduction strategy (Dixon et al., 2017), thus future research should examine quiet ego as a method of disrupting maladaptive perceptions (such as threat) associated with negative attitudes about Muslims. Interestingly, there was no significant indirect effect for external motivation to express prejudice, but there was for the internal motivation to express prejudice. This suggests quiet ego may be more effective in reducing internal vs. external pressures to express prejudice toward Muslims, though future research capable of making causal inferences is needed.

Moreover, although we found quiet ego was negatively associated with both internal and external motivations to express prejudice in Study 2, only internal motivation to express prejudice was directly associated with attitudes toward Muslims. This could be due in part to norms prohibiting the overt expression of prejudices, including toward Muslims. Crandall et al. (2018) found expression of prejudice toward Muslims (along with several other marginalized groups such as immigrants) was slightly more permissible following the 2016 U.S. presidential election, though this difference was not statistically significant. This suggests perceptions of social norms about the acceptability of prejudice toward Muslims are relatively stable. Additionally, data from Crandall et al.’s study shows beliefs about the acceptability of expressing prejudice toward Muslims (before and after the 2016 presidential election) were below the midpoint of the scale, suggesting people generally believe it is unacceptable to express prejudice toward Muslims. Together, this suggests that the external motivation to express prejudice toward Muslims may be low due to social norms against it, thus unrelated to attitudes toward Muslims. However, our sample in Study 2 was fairly liberal (although we ran the model controlling for political ideology). Future work may seek to test this association in a more ideologically diverse or conservative sample.

### Implications

There are three notable implications from this work: a contribution to the nomological network of quiet ego, contributions to the dual process model of ideology and prejudice, and potential applications to prejudice interventions. Regarding the nomological network, the relationships identified in our model support previously theorized mechanisms of quiet ego. Specifically, the ability to think less hierarchically about others supports the idea that quiet ego balances self and other concerns (*cf.*, Wayment and Bauer, 2017a). Further, mechanisms associated with growth-related experiences suggest an ability to eschew rigid political views by providing opportunities for new experiences and learning from individuals outside of one’s in-group. Recent studies investigating quiet ego characteristics have shown that even in stressful situations, quiet ego characteristics are associated with growth outcomes (Wayment et al., 2019a; Wayment and Silver, 2021). Additionally, recent work has identified quiet ego as a strong correlate of the light triad, a beneficent orientation toward others (Kaufman et al., 2019), further supporting the relationship between quiet ego and positive attitudes toward others. Thus, the results of our study provide additional evidence for the construct validity of the quiet ego (and scale) as well as insight into constructs associated with positive attitudes toward Muslims.

In Study 2, we found quiet ego was negatively associated with RWA and SDO which explained the association between quiet ego and attitudes toward Muslims. Notably, the dual process model of ideology and prejudice suggests RWA is in part influenced by greater perceptions of threat (Duckitt and Sibley, 2007). Those higher in quiet ego characteristics may perceive Muslims as less threatening, thus increasing the boundaries of intergroup acceptance. This could be particularly important for attitudes toward Muslims, as negative attitudes toward this group are often rooted in perceptions of threat (Velasco González et al., 2008; Wirtz et al., 2016), and competition (Schlueter et al., 2020), which underlie ideological orientations that promote prejudice such as RWA and SDO (Duckitt and Sibley, 2017). Additionally, threat may explain the association between quiet ego and positive attitudes toward other marginalized social groups who are subject to negative threat-related stereotypes (e.g., refugees and immigrants; Al-Kire et al., 2022). Because perceptions of threat and competition were not directly measured in our studies, subsequent research may seek to identify if such perceptions explain the associations between quiet ego and RWA and SDO.

Additionally, the dual process model of ideology and prejudice places RWA and SDO as two sets of social attitudes, which explain much of the variance in prejudice (Duckitt and Sibley, 2017). However, these social attitudes do not directly consider the internal and external motivations to express prejudice. In our model, we included internal and external MTEP to further understand attitudes toward Muslims and consider these motivational pressures. In doing so, we explained additional variance in attitudes toward Muslims, and extended the dual process model framework. Future research using the dual process model of ideology and prejudice may seek to further understand other motivations and contextual factors which further explain intergroup attitudes.

Although the design of our studies was correlational and incapable of assessing causality, the direction of our model is theoretically supported. As such, we can speculate about potential implications of this research for prejudice reduction techniques or intervention strategies, while recognizing future experimental or longitudinal work is needed to assess causality more directly. While some intervention research has targeted core personality traits (e.g., openness to experience; Sparkman et al., 2016; honesty-humility; Lee et al., 2010) and other individual difference variables (e.g., RWA and SDO; Ekehammar et al., 2004), these interventions may not maximize efficacy. To understand lay beliefs about the causes of prejudice, researchers found most participants cite closed-mindedness as the most important predictor of ethnic prejudice, and interventions facilitating open-mindedness and tolerance would be most efficacious in reducing prejudice (Miglietta et al., 2014). Given the robust associations between (low) openness, SDO, and RWA which underlie prejudiced attitudes (Sibley and Duckitt, 2008; Hodson et al., 2009), these reflect important intervention points. However, open-mindedness is a personality trait, which is relatively stable throughout the lifespan and not amenable to acute changes like quiet ego has been shown to be (Wayment et al., 2015). In line with our results, one suggestion that has been made in the past is to reduce threat and anxiety by increasing trust and empathy (Hodson, 2011). Given the relation between quiet ego and empathy, increasing quiet ego characteristics could further contribute to this pathway for potential prejudice interventions. For example, interventions that help strengthen universal and benevolent values may provide another avenue to reduce the types of thinking and justification associated with prejudice. More specifically, future work may seek to apply a quiet ego intervention to promote positive intergroup attitudes. Specifically, the quiet ego construct has been adapted into a brief quiet ego contemplation (QEC: Wayment et al., 2015), or short intervention. In a longitudinal study, the QEC intervention was related to several positive changes in first year college students, including increases in pluralistic thinking, perspective-taking, tolerance, openness to having views challenged and discussing controversial issues, and work cooperatively with diverse people. Additionally, a workshop based on the quiet ego was recently developed and tested and results indicated increases in compassion-related values (Wayment et al., 2019b). These examples could provide a good starting point for developing interventions aimed at reducing prejudice against Muslims, or other stigmatized groups.

### Limitations and additional future directions

Like all research, ours was not without limitation. First, our research design was cross-sectional, hindering our ability to make causal inferences. The work presented here represents a theoretically derived plausible model; however, there may be other theoretically consistent models. Future work should follow up on the mediation models presented in our studies and utilize experimental and longitudinal designs which can speak more directly to causal relations. Relatedly, there are likely other important mechanisms at play in the association between quiet ego and prejudice toward Muslims. For example, uncovering the mechanism by which the quiet ego facilitates identity fluidity or blurs the boundary between in-group and out-group would be extremely beneficial. Future work may seek to identify how quiet ego is associated with other variables and processes such as identification with all humanity or moral expansiveness, which both predict positive intergroup attitudes (Crimston et al., 2016; Dunwoody and McFarland, 2018). Moreover, given the evidence for a bidirectional relation between empathy and SDO (Sidanius et al., 2013), future work should also test whether similar bi-directional paths may apply to the relations tested in our model.

Interestingly, in Study 1, religious affiliation and race were unrelated to attitudes toward Muslims. The lack of significant associations between these characteristics and prejudice may have been because they do not capture strength of group identification or ideological attitudes which are more strongly related to intergroup attitudes. It is also possible that the dichotomous scoring of these variables masked potentially meaningful subgroup differences. For example, those with stronger Christian identities may demonstrate more prejudiced attitudes toward Muslims in an effort to maintain positive distinctiveness, consistent with social identity theory (Ysseldyk et al., 2010). However, such tests would have been underpowered in the current study, limiting the ability to make strong inferences. Future work may test these possibilities and examine subgroup differences alongside indices of ingroup identification in their associations with prejudice toward Muslims.

Additionally, data from Study 2 was collected from a college student sample. Although we established our basic associations between quiet ego and attitudes toward Muslims in Study 1, the full model may have been influenced in part by education level. Consistent with this idea, previous work on prejudice shows those who are more educated are often less prejudiced toward marginalized outgroups, including Muslims (Strabac and Listhaug, 2008). Future work may seek to test whether the results obtained in our studies are robust to education level.

Another important variable absent from our models in Studies 1 and 2 was knowledge of Islam and familiarity with Muslims. Previous work shows those with accurate knowledge of outgroups tend to be less prejudiced (Mansouri and Vergani, 2018). Additionally, consistent with the intergroup contact hypothesis, those who are more familiar with or who have close connections to members of outgroups tend to be less prejudiced (Pettigrew and Tropp, 2006). Future work may test whether our results are robust to the inclusion of knowledge of Islam and intergroup contact with Muslims.

Moreover, it is important to determine the relationship between quiet ego and other individual difference variables related to prejudice. For example, recent work has identified respect as a crucial component of out-group tolerance in a sample of highly conservative adults (Simon et al., 2019). Understanding how quiet ego is related to respect for out-group members (beyond just warmth or positive attitudes) could shed light on other relevant mechanisms. Additionally, it could be the case that quiet ego works by way of a common mechanism, or multiple pathways of influence may be involved. For example, the quiet ego characteristics of perspective-taking and inclusive identity tap directly into the idea that humans share important characteristics and are fundamentally interdependent. This empirical question should be addressed in future research.

Finally, it should be noted that researchers have identified similar patterns of prejudice toward outgroup members from both sides of the political spectrum (Brandt and Van Tongeren, 2017; van Prooijen and Krouwel, 2019). Although this study focused on attitudes among those oriented toward more conservative political ideologies (i.e., RWA and SDO), the process through which quiet ego is associated with positive intergroup attitudes should also be applicable to those who endorse stronger left-wing beliefs (e.g., left-wing authoritarianism and egalitarianism). However, this remains an empirical question which should be tested in future research. Future work may seek to test whether quiet ego is associated with positive attitudes toward more conventional or high-status groups, and if left-wing authoritarianism and egalitarianism (the opposite of SDO) similarly explain these relations.

## Conclusion

Overall, these results showed that quiet ego characteristics were positively associated with positive attitudes toward Muslims, both directly and indirectly through negative associations with individual differences associated with intergroup bias such as RWA, SDO, and internal MTEP. Moreover, these findings were robust to inclusion of other predictors of intergroup biases and replicated across two studies. These results further the understanding of the quiet ego construct by demonstrating its relation to attitudes toward Muslims. These findings also contribute to the intergroup relations literature by providing further insight into the predictors of positive intergroup attitudes. Given that the number of Muslims in the United States is growing at a steady pace (Pew Research Center, 2018), understanding the mechanisms of positive intergroup attitudes across marginalized outgroups and various cultural contexts would be an important and promising area for future empirical inquiry.

## Data availability statement

The raw data supporting the conclusions of this article will be made available by the authors, without undue reservation.

## Ethics statement

The studies involving human participants were reviewed and approved by Baylor University and Northern Arizona University. The patients/participants provided their written informed consent to participate in this study.

## Author contributions

RA-K conceived of study, collected and analyzed data, and wrote the original draft of the manuscript. HW conceived of study, analyzed data, and wrote portions of the manuscript. BE, KC, and J-AT wrote portions of the manuscript. KC also acquired funding for study 1. All authors contributed to the article and approved the submitted version.

## Funding

Our research in Study 1 was funded by the Henry Luce Foundation: The Luce Fund for Interreligious Theological Education Initiative.

## Conflict of interest

The authors declare that the research was conducted in the absence of any commercial or financial relationships that could be construed as a potential conflict of interest.

## Publisher’s note

All claims expressed in this article are solely those of the authors and do not necessarily represent those of their affiliated organizations, or those of the publisher, the editors and the reviewers. Any product that may be evaluated in this article, or claim that may be made by its manufacturer, is not guaranteed or endorsed by the publisher.

## Footnotes

^1^ https://IPhub.info

^2^In this paper, we specifically focused on attitudes toward Muslims. However, we also collected thermometer ratings for a series of other religious, racial, and ethnic groups (see Method). Participants higher in quiet ego reported warmer thermometer ratings for all target groups, including Muslims (*r*s 0.28 = 0.46, *p*s < 0.001).

^3^Data from (*n* = 2) Muslim-identifying participants were excluded from analyses.
